# Mitral and Tricuspid Valve Disease in Athletes

**DOI:** 10.3390/jcm12103562

**Published:** 2023-05-19

**Authors:** Andrea Segreti, Mihail Celeski, Luigi Maria Monticelli, Alfonso Perillo, Simone Pasquale Crispino, Giuseppe Di Gioia, Valeria Cammalleri, Chiara Fossati, Simona Mega, Rocco Papalia, Fabio Pigozzi, Gian Paolo Ussia, Francesco Grigioni

**Affiliations:** 1 Unit of Cardiovascular Science, Fondazione Policlinico Universitario Campus Bio-Medico, Via Alvaro del Portillo, 00128 Roma, Italy; 2 Department of Movement, Human and Health Sciences, University of Rome “Foro Italico”, Piazza Lauro de Bosis, 00135 Roma, Italy; 3 Institute of Sports Medicine, Sport and Health, National Italian Olympic Committee, Largo Piero Gabrielli, 00197 Roma, Italy; 4 Research Unit of Orthopaedic and Trauma Surgery, Department of Medicine and Surgery, Università Campus Bio-Medico di Roma, Via Alvaro del Portillo, 00128 Roma, Italy; 5 Research Unit of Orthopaedic and Trauma Surgery, Fondazione Policlinico Universitario Campus Bio-Medico, Via Alvaro del Portillo, 00128 Roma, Italy

**Keywords:** mitral valve disease, tricuspid valve disease, cardiac adaptation, athletes, sports activity

## Abstract

Observing mitral or tricuspid valve disease in an athlete raises many considerations for the clinician. Initially, the etiology must be clarified, with causes differing depending on whether the athlete is young or a master. Notably, vigorous training in competitive athletes leads to a constellation of structural and functional adaptations involving cardiac chambers and atrioventricular valve systems. In addition, a proper evaluation of the athlete with valve disease is necessary to evaluate the eligibility for competitive sports and identify those requiring more follow-up. Indeed, some valve pathologies are associated with an increased risk of severe arrhythmias and potentially sudden cardiac death. Traditional and advanced imaging modalities help clarify clinical doubts, allowing essential information about the athlete’s physiology and differentiating between primary valve diseases from those secondary to training-related cardiac adaptations. Remarkably, another application of multimodality imaging is evaluating athletes with valve diseases during exercise to reproduce the sport setting and better characterize the etiology and valve defect mechanism. This review aims to analyze the possible causes of atrioventricular valve diseases in athletes, focusing primarily on imaging applications in diagnosis and risk stratification.

## 1. Introduction

Mitral valve (MV) and tricuspid valve (TV) diseases can be commonly observed in athletes [1]. However, identifying the etiologic cause is essential and requires carefully evaluating suitability for competitive activity and specific concerns about the appropriate management. In addition, the etiology of valve diseases may vary depending on the athlete’s age. Indeed, it may be a congenital or genetic syndrome or a complication of acquired rheumatic fever. Nevertheless, a primarily degenerative disorder is the most frequently responsible cause of valve abnormalities among master athletes [2].

Primary valvular heart diseases (VHDs) show similar prevalence in athletes and the general population; however, athletes are more likely to experience secondary (functional) mitral regurgitation (MR) and tricuspid regurgitation (TR) [3]. This observation may be partially attributed to the adaptive changes of atrioventricular remodeling in the context of the “athlete’s heart” [2,4]. Although many studies have focused on the adaptations of the cardiac chambers to physical exercise, the effects of exercise on MV and TV should be clarified. In addition, besides identifying valve disease etiology, an essential aspect to consider when facing athletes with valve disease is whether the cardiac chamber enlargement is secondary to exercise-induced cardiac remodeling (EICR) or underlying valve disease.

VHD can reduce athletes’ performance depending on the severity degree. Generally, right-sided valvular abnormalities are better tolerated than left-sided valvular abnormalities, and regurgitant lesions are better tolerated than stenotic lesions [5]. Another essential aspect to consider is whether athletes with MV and TV disease are suitable for sports activity, mainly for those disciplines requiring high-intensity and increased cardiovascular (CV) burden during exercise [6]. Generally, asymptomatic athletes with mild valvular disease, normal ventricular function, and suitable functional capacity can engage in all competitive sports. However, in the case of more than a mild valvular abnormality, the suitability for sports should be personalized, considering additional parameters (Table 1). Therefore, correctly identifying those athletes requiring treatment is a necessary step contributing to safer sports participation.

In this complex scenario, traditional and advanced imaging modalities, including echocardiography, cardiac computed tomography (CCT), and cardiac magnetic resonance (CMR), are helpful in non-invasively differentiating several etiologies and better identifying athletes at risk of arrhythmias and indicating whether the athlete needs a particular treatment and if they can engage in a specific competitive sport.

## 2. Training-Induced Atrioventricular Valve Remodeling in Athletes

Remarkably, it has been demonstrated that athletes with structurally normal valves without a known cardiac disease have an overall higher statistically significant prevalence of functional valve regurgitation rate than non-athlete controls (91% vs. 38%, *p* < 0.001). In particular, MR prevalence was 69% vs. 27%, while TR prevalence was 76% vs. 15% [7].

An athlete is defined as an individual of young or adult age, either amateur or professional, who is engaged in regular exercise training and participates in official sports competitions [6]. As stressed by the European Society of Cardiology guidelines on CV prevention, regular aerobic physical activity has several favorable CV benefits. [8]. However, most elite and competitive athletes (CAs) exercise above the recommended limits and regularly engage in more than 20 h of intense weekly activity. It is well known that prolonged and drastic physical activity exposes the heart to significant hemodynamic pressure and volume overload stresses. The results are specific CV electrical, structural, and functional changes, commonly identified as “athlete’s heart” [9,10], aimed to increase the O_2_ supply to the working muscles during exercise [11].

The degree of EICR may be marked and influenced by several factors [2,11], including individual characteristics, environmental factors, the type of sports discipline participated, the role of the athlete, and the degree of hemodynamic stress. [9,10] Athletic training increases cardiac chamber sizes and left ventricle (LV) wall thickness, with different degrees depending on whether the athlete performs a prevalently static (e.g., weightlifting), dynamic (e.g., cycling), or a mixture of components (e.g., soccer). Accordingly, a more inclusive classification of sports disciplines foresees the relative isometric (or static) and isotonic (or dynamic) components and includes four groups: skill, power, mixed, and endurance [6,9,10,12].

The CV system adaptation to training includes changes in stroke volume (SV), heart rate (HR), blood pressure (BP), peripheral vascular resistance, LV afterload, cardiac chambers remodeling, and sympathetic/parasympathetic balance [9,10,13,14]. In particular, endurance sports require an extensive and prolonged 5- to 6-fold cardiac output (CO) increase to sustain high workloads at a submaximal effort over protracted periods with resulting higher peak O_2_ consumption [15,16]. Indeed, endurance sports are characterized by eccentric remodeling. On the contrary, power activity exposes the CV system to increased BP levels during bursts, leading to a chronic adaptation characterized by concentric remodeling [9,11].

Therefore, a balanced adaptation of the cardiac chambers is a characteristic and unmistakable feature of an athlete’s heart. However, although ventricular and atrial dilation can significantly affect atrioventricular annular geometry and related valvular competency in various pathological states, less is known about the EICR alterations in the shape and function of the mitral annulus (MA) and tricuspid annulus (TA) in CAs [4,17].

The MA and TA are elliptical, saddle-shaped, and dynamic structures that connect the leaflets with the ventricular and atrial myocardium. Elite athletes have a significant dilatation of the MA and TA dimensions and an increase in the tenting of both atrioventricular valves compared to non-athlete counterparts, [4] indicating that atrioventricular annuli undergo disproportionate remodeling in response to regular training. [4] Indeed, through a combination of annulus enlargement and tethering of the valve leaflets, functional regurgitation of the MV or TV has been primarily linked to LV or right ventricle (RV) remodeling, respectively [1].

Therefore, adaptation to exercise determines several changes, including atrioventricular remodeling, with endurance athletes demonstrating the highest increase in cardiac chambers and atrioventricular remodeling. However, the abrupt surge in BP characteristically associated with a burst of muscle contraction in power sports may also contribute to atrioventricular valve disease. Nevertheless, several characteristics of the athlete’s heart could be related to the higher prevalence of regurgitation in the atrial and ventricular functional remodeling, cardiac chamber size increase, slower HR, increased SV, and altered RV and LV inflow patterns [7].

Despite the ventricular functional regurgitation, it is now widely known that there is a type of atrioventricular regurgitation in patients with structurally intact leaflets, normal geometry and function of the ventricles, and dilated atria, called atrial functional regurgitation [18,19]. Many characteristics of both forms of functional regurgitation, such as annular enlargement and increased leaflet tenting, are present in the athlete’s heart; in particular, a significant atrial and ventricular dilatation may magnify the effect on annular geometry and valve function. Hence, a mixed form of functional regurgitation might be assumed in athletes with MR or TR [4].

As claimed before, in isometrically conditioned athletes, there is a significantly greater degree of ventricular hypertrophy, i.e., a greater relative wall thickness (RWT) and less outward displacement of the papillary muscles (PMs). Furthermore, this LV response to exercise is less likely to tether the MV and less likely to cause significant MR [20]. These features indicate that healthy athletes engaged in isometric sports, i.e., characterized by pressure overload and LV concentric hypertrophy [21], have less displacement of the PMs outward. On the contrary, specific mitral abnormalities may be present in athletes affected by hypertrophic cardiomyopathy (HCM); these alterations include elongation of mitral chordae with systolic anterior motion and flow acceleration in the LV outflow tract (LVOT), a phenomenon not described in otherwise healthy athletes [22,23].

The described physiological or pathological features can be appropriately identified by several imaging modalities, including different echocardiographic applications, CCT, and CMR [21,22,23]. In particular, Table 1 describes the possible applications of advanced imaging modalities in evaluating athlete’s valve diseases.

## 3. Multimodality Imaging Assessment of Mitral and Tricuspid Valve Disease in Athletes

Imaging is pivotal in evaluating athletes with valve diseases and identifying those at increased CV risk when exercising [24]. Indeed, managing athletes with VHD requires a structured approach incorporating several paramount factors; these include the presence of symptoms, functional capacity, characteristics of the valvular lesion, impact on cardiac chambers, and effect on pulmonary artery pressure (PAP) [5].

Accordingly, accurately interpreting cardiac imaging in an athlete, explicitly differentiating EICR from pathologic hypertrophy, requires careful consideration of a specific athlete’s predominant sporting discipline [2,24]. Another critical aspect to consider is whether an athlete presents a larger chamber size and wall thickness and, eventually, if it is a physiologic response in the context of EICR or depending majorly on a valve regurgitation. In addition, it must be clarified if atrioventricular remodeling in the context of EICR is associated with an increased incidence of valvular regurgitation [2]. Indeed, in the presence of LV cavity dilation, the specific impact of MR should be interpreted in the context of the athlete’s type of sport and considering the body size [21].

The classical ventricular type of functional regurgitation is mainly characterized by increased leaflet tenting, whereas a notably enlarged annulus refers to atrial functional regurgitation. Notably, the athlete’s heart shares several features of both types of functional regurgitation; in addition, pronounced ventricular and atrial dilation may exaggerate their impact on annular geometry and valvular function. Thus, a mixed type of functional regurgitation can be assumed in athletes with MR or TR [4]. Although these regurgitations are minor, they will result in a larger pendulating volume of blood, which may increase the stretching force in the atria [3]. For instance, athletes with MR have a higher MA and TA and a more pronounced saddle shape of the MA than those without MR [4].

Furthermore, the sport may trigger life-threatening ventricular arrhythmias (VAs) and sudden cardiac death (SCD) in predisposed patients with structural or electrical abnormalities; hence, the differentiation of physiological from pathological remodeling by imaging is a fundamental step [25]. Although the ventricular myocardium’s dynamic adaptation is the most widely known aspect when considering an athlete’s heart, alterations of the atrioventricular valve apparatus should not be overlooked [4].

A broad spectrum of imaging modalities is available to evaluate the athletes, allowing the clinician to adopt a step-by-step and comprehensive approach [22].

### 3.1. Resting Echocardiography

Transthoracic echocardiography (TTE) is the CV imaging method of choice in the athletic population by its widespread availability, relatively low cost, and safety.

In most cases, in athletes, TTE is a second-level examination following a physical examination and a 12-lead electrocardiogram. Different echocardiography applications are available, from traditional two-dimensional echocardiography (2DE) to three-dimensional echocardiography (3DE).

Indeed, in most athletes, the low acoustic impedance of the chest makes it possible to obtain high-quality images and assess all the cardiac structures [26]. In particular, TTE can provide complementary information on cardiac morphology, functional properties, and hemodynamics, playing an essential role in diagnosing and managing a broad spectrum of CV conditions, including VHDs [26]. Indeed, TTE allows a direct evaluation of the structure and function of the atrioventricular valves and a comprehensive evaluation of the subvalvular complex. In addition, TTE provides data regarding the severity of valve failure, cardiac function, PAP, and other hemodynamic consequences [13,27,28,29].

EICR is the term used to describe the unique modifications in heart structure and function that result from regular physical activity, with dichotomous LV remodeling patterns described by Morganroth et al. [30]. Generally, in athletes practicing endurance sports, the volume challenge posed by prolonged, repeated increases in CO causes biventricular dilatation and differential increments in left ventricular (LV) wall thickness or eccentric LV hypertrophy defined by increased LV mass and a RWT < 0.42 [31]. On the other hand, strength training increases the pressure load on the LV and aorta, causing LV concentric hypertrophy (LV thickening without significantly increasing the size of the LV chamber), defined by increased LV mass and a RWT > 0.42 [24]. Generally, LV wall thickness > 13 mm and LV diameter > 60 mm are rarely found in athletes without heart disease [32]. However, in a study of 1309 Italian elite athletes representing 38 different sports, the LV end-diastolic dimension (LVEDD) was more than 55 mm in 45% and more than 60 mm in 14% of the participants, which, according to other studies, would misclassify nearly 40% of the athletes as pathological [32]. As athletes frequently have significant physiologic dilation, it is not advised to utilize precise cut-off values for LVEDD or LV end-diastolic volume (LVEDV) alone to distinguish between EICR and cardiomyopathy [24]. In addition, in athletes, cardiac function should not only be evaluated by the “classic” measurement of LV ejection fraction (EF) (LVEF). Indeed, other methods, such as global longitudinal strain (GLS) and myocardial work (MW), represent a better indicator of LV contraction in athletes and may increase the potential for myocardial pathology, especially in the presence of questionable LV hypertrophy or enlargement [22,33,34]. If a 2D resting echocardiogram yields conflicting results, exercise echocardiography and 3DE may provide valuable additional data needed to assess the severity of valve impairment [1].

Early echocardiographic results reported that CAs, especially endurance athletes, frequently have an enlarged left atrium (LA), with LA diameter being larger than 4.6 mm in endurance CA, 3.5 mm in mixed strength and endurance CA, and 2.9 mm in just strength trained CAs when compared to inactive controls [35]. LA size is generally measured as LA diameter, LA area from a 4-chamber view, and LA volume by biplanar apical 2- and 4-chamber views. While measuring LA diameter underestimates LA dimensions, measuring LA volume more accurately represents LA’s dilation. For example, when the upper LA diameter cut-off was placed at 40 mm, research by Pelliccia et al. found that 20% of cases of enlarged LA size were present among the athletes [36]. However, a recent study proposes 57 mL/m^2^ as the maximum expected value for LA indexed volume in CA and the mean + 2 deviation standards to distinguish from pathological values [3].

In addition, recent evidence claims a higher end-systolic wall stress for the RV than the LV due to a higher relative increase in systolic PAP (sPAP) compared to systolic arterial BP during endurance exercise, which is associated with pressure and volume load [37]. Indeed, the increased necessity of oxygen during exercise and biventricular CO, along with the higher decrease in pulmonary vascular resistance than systemic resistance, leads to an abnormal increase in PAP and RV afterload [38]. Another determinant of RV afterload is the significant atrial filling and rise in pressure during systole due to the high-flow state exercise related. All these factors lead to progressive RV enlargement [38]. Indeed, intense endurance exercise can provoke RV dysfunction, defined by tricuspid annular plane systolic excursion (TAPSE) < 17 mm, S’ < 9.5 cm/s, or fractional area change (FAC) < 35%. However, RV dysfunction can recover after a short time of detraining, although some athletes can show chronic anatomical alterations and diminished right ventricular function [25]. Commonly, in athletes, there is mild to moderate RV dilatation during the remodeling without significant RV hypertrophy, with nearly 15% exceeding a basal RV dimension of 40 mm, as demonstrated in the study of D’Andrea et al. [39]. Nevertheless, concurrent dilatation of other cardiac chambers is always present in an athlete with a physiologic dilation, along with intact RV systolic function, normal or improved LV diastolic function, symmetric LV hypertrophy with only mild segmental variation, preserved or low normal resting systolic function, and preserved LV systolic strain indices [22,24]. Indeed, an abnormal isolated RV dilatation in an athlete raises the possibility of underlying heart disease [24].

Recent data confirm that atrial adaptation on exercise also involves the right atrium (RA). It has been shown that athletes have larger RA dimensions than non-athletes, with RA area being even more significant among those athletes who present TR than athletes without [3]. Since RA shape is asymmetrical and cannot be recorded from a biplane, it should be evaluated as an area utilizing the apical 4-chamber view. On the other hand, RA volumes are underestimated with 2DE techniques compared with 3DE [40]. According to Gjerdalen et al., the highest limits for athletes should be 14.5 cm^2^/m^2^ for the RA area and 2.9 cm/m^2^ for the RA minor axis [3]. Regarding atrial function in athletes, there have been inconsistent findings from studies that have used speckle-tracking echocardiography to measure atrial function [41,42]. In addition, due to atrial dilatation, adult athletes experience a greater risk of supraventricular arrhythmias [36].

All these anatomic and functional cardiac chamber modifications can be the cause, or the consequence, representing an additional echocardiographic challenge in evaluating valve abnormalities. Therefore, in patients where TTE is inconclusive or technically challenging or when surgical intervention is considered, knowledge of the specific MV and TV anatomy is necessary, which can be obtained with trans-esophageal echocardiography [13].

Mitral valve prolapse (MVP) at echocardiography, in parasternal long axis or apical 3-chamber view, is characterized by a systolic billowing of any portion of the mitral leaflets more than 2 mm past the mitral annular plane [2]. On color Doppler, MR in the context of MVP is characterized by a modest proximal flow convergence area at early systole, which grows throughout mid-systole and often reaches a maximum in late systole [43]. Moreover, leaflets are commonly lengthened and thickened. A typical (i.e., classic) MVP is characterized by a leaflet thickening of more than 5 mm as measured in the parasternal long-axis view in tele-diastole [44]. In addition, 2-D echocardiography and speckle tracking can show annulus enlargement associated with annulus flattening, mainly basal LV dilatation, and decreased contraction in MVP, which contribute to the progression of the degenerative process [45,46]. Therefore, myocardial stretch caused by the prolapsing leaflets leads to increased tissue velocity that sharply affects the PMs and myocardium and can be assessed by tissue Doppler imaging from the apical view, with the probe positioned in a parallel line with the MA motion [47]. Muthukumar et al. suggested the application of the Pickelhaube sign, which is an increase in tissue Doppler systolic wave peak velocity at the lateral MV of 16 cm/sec or more; this sign is regarded as an indicator for the arrhythmic MVP and identifies individuals who might show late gadolinium enhancement (LGE) on CMR [48].

Regarding MR, as mentioned, echocardiography is crucial in determining etiology, morphology, and mechanism, especially in secondary or functional origin cases. Therefore, many echocardiographic methods should be considered to quantify MR [27,49,50,51,52], as reported in Table 2. The updated 2020 American College of Cardiology/American Heart Association Guidelines report that threshold criteria for functional MR should now be similar to that for primary MR, i.e., effective regurgitant orifice area (EROA) ≥ 0.4 cm^2^, regurgitant volume (RVol) ≥ 60 mL, and a regurgitant fraction (RF) ≥ 50% [53]. Similarly, the 2021 European Society of Cardiology/European Association for Cardio-Thoracic Surgery Guidelines maintained the same cut-offs, adding an MR severity confirmed for EROA ≥ 0.3 cm^2^ in an elliptical regurgitant orifice area and RVol ≥ 45 mL in low flow conditions [27]. However, the quantification of functional MR using the EROA method has been a topic of much controversy and great debate. Based on the results of the MITRA-FR [54] and COAPT [55] trials, Grayburn et al. reported that the ratio of EROA to LVEDV end-diastolic volume is probably of value in each patient’s clinical decision-making, as patients with proportionate MR may be strongly prone to responding to the optimal medical and device therapy; in contrast, patient with disproportionate MR might benefit from additional mitral transcatheter repair [51]. However, it is not known whether the exercise-induced LV dilatation in endurance athletes may contribute to the “disproportionality” among those with severe MR and whether it necessitates a revaluation of the cut-offs before deciding on a transcatheter repair.

TTE also allows a comprehensive evaluation of TR severity [52], as reported in Table 2. Indeed, the density and shape of regurgitant signal on color flow, in addition to quantitative parameters such as vena contracta (VC) width, proximal isovelocity surface area (PISA), early diastolic filling velocity, RV outflow tract (RVOT) velocity of TR, EROA, and RVol, are established echocardiographic parameters to assess the severity of TR [1,56]. However, the PISA approach may underestimate the degree of TR, and if conflicting results are obtained, the 3D VC area may be assessed [10]. In addition, no isolated Doppler and echocardiographic measurements or parameters are precise enough to quantify TR severity, and integration of multiple parameters is required. When multiple parameters are concordant, TR grade can be determined with high probability (especially for mild or severe TR) [52]. Moreover, over the years, a new TR grading scheme, which shows a predictive value, has been proposed to categorize the severity of TR better, including “massive” TR (VC 14–20 mm, EROA 60–79 mm^2^, 3D VC area or quantitative EROA 95–114 mm^2^) and “torrential” TR (VC ≥ 21 mm, EROA ≥ 80 mm^2^, 3D VC area or quantitative EROA ≥ 115 mm^2^) [57]. In addition, according to Izgi et al., the RV early inflow–outflow (RVEIO) index, calculated as the difference between the early diastolic filling velocity (E wave) and the RVOT velocity time integral (VTI) (RVEIO index = E wave velocity (cm/s)/RVOT VTI (cm)) is a straightforward, reliable, and independent predictive factor of severe TR that complements existing techniques for determining TR severity [58].

Table 2 summarizes several methods to measure and quantify atrioventricular valve diseases, differentiating between athletic and non-athletic populations.

### 3.2. Exercise Stress Echocardiography

Exercise stress echocardiography (ESE) is a non-invasive diagnostic test that is trustworthy, secure, and can help identify cardiac pathologic conditions among athletes by reproducing sports practiced [22].

For example, athletes with LV hypertrophy reporting symptoms such as dyspnea or syncope with echocardiographic LVOT gradient higher than 50 mmHg during or right after exercise can be affected by HCM [59]. ESE is also helpful in evaluating the contractile reserve during physical activity; its significant improvement during exercise indicates that ventricle enlargement is part of the physiological EICR [22]. Indeed, athletes with physiologically enlarged LV volume and borderline LV global function may be unnecessarily removed from competition if incorrectly diagnosed with dilated cardiomyopathy (DCM). In particular, during ESE, the inability to improve the LVEF > 11% from rest to peak exercise and failure to elevate the LVEF > 63% at peak exercise are indicative of DCM, with 80% of sensitivity and 90% of specificity [60]. Furthermore, for determining functional status, hemodynamic changes during exercise (LV filling pressure, PAP), valvular functional parameters (transvalvular gradients, regurgitation entity), presence of induced myocardial ischemia or arrhythmias, all athletes with mild to moderate VHD should undergo an exercise stress test and ESE using a protocol that slightly approximates the amount of physical effort predicted from the sport they wish to participate in [1].

In general, exercise produces no significant change or a mild decrease in the RF because of reduced systemic vascular resistance. However, patients with elevation of HR (increased systolic ejection time per minute) or BP with exercise may manifest marked increases in Rvol and pulmonary capillary pressures [2].

In patients with asymptomatic mitral stenosis (MS), exercise testing can be beneficial by revealing latent symptoms. In addition, ESE helps determine whether symptomatic patients with only mild or moderate MS have provoked high transvalvular gradients or inducible pulmonary hypertension (PH) by Doppler echocardiography assessment, determining how much exercise is permissible [61]. For example, CA who experience a significant rise (i.e., >40 mmHg) in sPAP during exercise are more likely to experience long-term adverse effects on RV function [1]. In addition, during exercise, the abnormal response identified as an increase in the mean transmitral gradient ≥15 mmHg or estimated sPAP ≥60 mmHg is a sign of significant MS [62].

Moreover, ESE gives essential information in patients with valve prostheses because it detects hemodynamic changes when the mean transvalvular gradient increases disproportionately during exercise (>20 mmHg for aortic prostheses or >12 mmHg for mitral prostheses), which may indicate severe prosthesis stenosis or a significant patient-prosthesis mismatch [59].

On the other hand, the role of ESE in patients with MVP founds place, primarily when associated with primary MR. Elevated sPAP (>60 mmHg) during activity could be significant in CA with severe primary MR that is not showing any symptoms. In addition, failure of LVEF to improve regularly with exercise indicates poorer postoperative LV function [63]. Furthermore, assessing LV contractile reserve, mainly through evaluating exercise-induced changes in LV myocardial longitudinal function, may improve risk stratification and clinical decision-making in patients with MVP [64]. In athletes with moderate MR, a stress test during exercise is advised to assess functional ability, hemodynamic response, and complex arrhythmias that, when negative, provide eligibility for participating in competitive sports [1]. It is widely known that the degree of MR severity can be dynamic, load-dependent, or elevated with physical activity [65]. Indeed, progressive exercise-induced increase in MR severity (EROA ≥ 10 mm^2^ and RVol ≥ 15 mL) can be found in nearly 30% of the patients, with a magnitude associated with variation of exercise-induced sPAP [66]. In addition, the absence of LV contractile reserve, i.e., > 5% increment in EF or less than −2% rise in GLS, the increment in regurgitation severity (at least 1 grade), exercise-induced PH (exercise sPAP ≥ 60 mm Hg), and the RV’s inability (measured by TAPSE < 19 mm) to handle the excessively high PAP during exercise are parameters of poor prognosis in asymptomatic patients with severe primary MR [59]. Furthermore, exercise-induced variations in functional MR severity are linked to modifications in LV remodeling and valvular deformation [67], and ESE may offer valuable data in patients with the following conditions: exertional dyspnea that is unrelated to the degree of resting LV dysfunction or MR severity; recurrent and unspecified acute pulmonary edema; obscured high risk of mortality and HF (increase in MR severity; EROA 13 mm^2^; sPAP > 60 mmHg), moderate MR before surgical revascularization, persistent PH following surgery [59].

Moreover, ESE may be used to evaluate the TV to uncover latent symptoms, discover inducible ischemia, and evaluate for latent or progressive valve dysfunction [68]. In particular, TAPSE, RV S’ wave, and FAC variations can be used to measure the RV’s functional reserve at rest and during stress; the inability to increase these indices suggests a decreased RV functional reserve [68]. Furthermore, with intermediate sensitivity and specificity, TR Vmax determined sPAP of > 45 mmHg or an increase of > 20 mmHg during low-intensity exercise suggests latent PH; however, attention should be made to athletes as they may achieve a sPAP of 55–60 mmHg during exercise [69].

### 3.3. Three-Dimensional Echocardiography

3DE enhances the diagnostic power of cardiac ultrasound in athletes with valve disease as it helps evaluate the cardiac chambers’ size and anatomy, ventricular function, valvular morphology and dynamics, and blood flow velocity [22]. Indeed, 3D planimetry has been described as the most precise technique for measuring the MV and TV area, characterizing MV and TV morphology and function, and quantifying and assessing MS and tricuspid stenosis (TS) [70,71]. In addition, 3DE allows clinicians to indicate whether the atrioventricular annuli undergo remodeling in response to regular exercise [4].

Regarding MVP, the techniques of 3DE, such as simultaneous multiplane mode, real-time 3DE, and ECG-triggered multi-beat 3DE (zoom and full volume mode), assist in identifying MV billowing and prolapse, identification of the etiology process and determination of the prolapsing or flail scallops [72]. Furthermore, 3DE makes it easier to identify the location and size of MV defects effectively, assess the MA geometry and biomechanics, examine dynamics of the annulus during MVP, and characterize the relationship between mitral annular disjunction (MAD) and paradoxical dilatation and systolic flattening of annulus [73,74].

Nowadays, the role of 3DE is becoming more critical in evaluating MR and TR. Indeed, it provides a more comprehensive evaluation of both MV and TV apparatus compared to 2DE. In addition, the total amount of the LA volume may not be captured by 2DE computations, and 3DE has more precision for LA assessment than 2DE [75]. Similarly, in atrial functional TR (AFTR), the RV takes on a triangular shape with dilatation restricted to the inflow basal region; hence measuring it just by RV basal diameter may provide the incorrect impression that it is more prominent. The sole technique permitting a valid measurement of RV dimensions is 3DE-derived RV volume, and RV EF evaluated by 3DE allows the categorization of patient prognosis [76]. According to a recent 3D echocardiographic study, regardless of heart rhythms or RV dynamic loading, RA volume is the most significant factor in the TA area. RA dilation is a crucial component of TA dilatation in functional TR [17]. Furthermore, by allowing direct planimetry of the VC regardless of orifice shape or number of jets in MR or TR, 3DE overcomes the assumptions of a circular regurgitant orifice section [77]. In the severe TR, studies proposed thresholds are 3D VC area ≥ 0.60 cm [2], Doppler-EROA ≥ 0.65 cm^2^, and PISA-EROA ≥ 0.34 cm [78]. Furthermore, it also aids in more precise measuring of coaptation depth, tenting area, the angle subtended by posterior MV leaflet, and a better understanding of the MA and TA geometry and dynamics. Moreover, for leaflet tethering assessment to distinguish ventricular functional mitral regurgitation (VFMR) and ventricular functional tricuspid regurgitation (VFTR) from their respective atrial equivalents and as a better indicator of regurgitation severity, the 3D tenting volume may be a more reliable measure [17].

All the described factors contribute to the differentiation between physiological and pathological modifications. For instance, athletes with MR displayed a more prominent MA enlargement, and this subgroup also revealed a more prominent TA remodeling, according to Fábián et al. [4]. In addition, athletes without MR had a more noticeable MA saddle shape, increased annulus height to commissural width ratio, and a less acute nonplanar angle. As chamber shape and intracardiac pressures continuously change during various exercise intensities, this final alteration may act as an adaptive adjustment to maintain correct coaptation [4].

### 3.4. Myocardial Work

Newer echocardiographic applications include the MW assessment, which seems to be a more reliable measure of myocardial contractility and contractile reserve in athletes than traditional echocardiographic measurements [79,80,81].

Indeed, studies evaluating 2D-derived GLS measured in endurance athletes at rest provided contrasting results, showing in some studies higher values, in others lower ones, or no differences with non-athletes [33]. This discordance may be related to the impact on the diagnostic utility of GLS strain measurements of multiple factors such as preload, afterload, LV geometry, and sinus bradycardia [33,82,83]. However, these limitations could be overcome by measuring GLS in athletes during exercise [84].

In addition, to exceed the load dependency and other limits of GLS, the MW has emerged as an alternative instrument for evaluating myocardial function [33]. MW can be obtained non-invasively through LV pressure-strain loop (PSL) analysis, which results from the non-invasive estimation of LV pressure (brachial blood pressure by a cuff) and strain analysis by STE [85]. This evaluation is crucial for athletes whose BP and loading conditions vary between exams and across distinct phases of their training regimen [22].

Therefore, in the currently available echocardiographic software, after calculating GLS in two-, three-, and four-chamber views, the values of brachial blood pressure and the time of valvular events, it is possible to obtain PSL and more precise information about myocardial contraction during the different phases of the cardiac cycle [85].

In particular, the determinants of MW indices that are produced by the software include global work index (GWI), global constructive work (GCW), global wasted work (GWW, mmHg%), and global work efficiency (GWE, %) [79,81].

GWI, expressed as mmHg%, is the amount of MW performed by the LV during systole obtained with the area of the PSL from MV closure to MV opening. GCW, expressed as mmHg%, is the positive work performed during myocardial shortening in systole and negative work during lengthening in isovolumic relaxation. GWW, expressed as mmHg%, is the negative work performed during lengthening in systole and positive work performed during myocardial shortening in isovolumic relaxation. Finally, GWE, expressed as %, is the relationship between constructive work and the sum of constructive and wasted work [79,81].

It was recently reported that LV adaptation in endurance athletes resulted in preserved GWE and GWW, even though with decreased values of GLS. In addition, GWE at rest can predict better than LVEF, functional capacity, and pulmonary or hemodynamic congestion individuated at peak exercise [81].

Therefore, the utility of MW indices is to provide a comprehensive insight into myocardial contraction, adding essential information to differentiate between physiological remodeling or pathological remodeling, for example, due to valve regurgitation. In this context, MW indices are pivotal in the clinical decision-making process of selecting patients that need valve surgery. For example, in the MVP, energy demand and oxidative stress are increased due to localized hypercontractility and recurrent segmental myocardial traction, which also causes localized myocyte hypertrophy and fibrous tissue repair [86]. For the identification of higher mechanical work and segmental hypercontractility, advanced TTE techniques can be applied, such as the segmental longitudinal strain of the mid-basal segments of the posterior and lateral walls [87]. The MW combines both longitudinal deformation and afterload. In addition, the most recent NORRE study offers helpful 2D TTE reference ranges for emerging non-invasive MW measures, which can guide the identification of the higher mechanical work [88].

Nevertheless, Ermakov et al. demonstrated an increase in mechanical dispersion in patients with MVP, especially in the arrhythmic MVP, compared with controls (52 vs. 42 ms, *p* = 0.005) despite the comparable values of LVEF and GLS [89]. Furthermore, a recent study [90] showed that LV MW characteristics connect with secondary MR grades. As the secondary MR degree worsened, the LV GWI and GCW became more compromised. Nevertheless, the LA is a low-pressure chamber; thus, emptying the LV into it may benefit myocardial energetics as demonstrated by the less impaired LV GWW while having a much worse degree severity regurgitation grade in the study of Yedidya et al. Moreover, their study showed a substantial correlation between LV MW indices and prognosis, with reduced LV GWW and impaired LV GWI and GCW being independently linked to all-cause mortality [91].

In the TV disease assessment, the evaluation of RV function plays a significant role. Therefore, RV MW (RVMW) is a new marker used to quantitatively assess RV function by incorporating the RV GLS (RVGLS), PAP, and tricuspid and pulmonic valvular events [91]. Wu et al. have recently discovered that RVMW indicators and RVGLS show robust linkages and may serve as credible indicators of RV myocardial systolic performance [92]. However, further studies are necessary to evaluate better the role of MW and its utility in clinical practice in athletes with valve disease.

### 3.5. Cardiac Magnetic Resonance

CMR is the most useful imaging technique for differentiating between physiology and pathology in athletes [22,24,93]. CMR provides precious information about the definition of myocardial morphology, wall motion assessment, heart chambers volumes, mass, functioning, tissue characterization, and more [22].

An athlete’s heart can be distinguished from cardiomyopathy using recent techniques such as LGE, native T1, extracellular volume (ECV) mapping, and T2 mapping [22].

As reported above, some MV or TV diseases are associated with increased SCD; therefore, CMR has a primary role in MV and TV evaluation, identifying myocardial subvalvular scarring and other factors related to increased risk of SCD and requiring preventive strategies such as specific management or appropriate sports restriction [93].

For example, in patients with MVP, fibrosis of the PMs and inferobasal LV wall, suggesting a myocardial stretch by the prolapsing leaflet, is the structural hallmark and correlates with VAs origin [86]. In particular, in athletes with MVP, CMR offers accurate details on the presence and extension of fibrosis, MV features, thickness and severity of leaflet prolapse, MAD and curling detection, regional hypertrophy, and myocardium strain, helping in the stratification for malignant VAs [94,95,96]. Moreover, CMR offers quantitative measurements of chamber size, Rvol, and RF in addition to other data on the MR process and myocardium viability [97]. However, even in patients with MVP and mild MR, the CMR can identify the existence of macro-fibrosis. Kitkungvan et al. demonstrated that replacement fibrosis is more represented among individuals with MVP than those without (36.7% vs. 6.7%; *p* < 0.001), and it is associated with a higher arrhythmic event rate [98].

In MVP patients, the most frequently individuated non-ischemic fibrosis pattern was mid-wall striae and patchy pattern of the basal inferolateral wall (31.1%), followed by the inferior basal wall (10.7%). These patterns differ from the typical LGE sub-endocardial or transmural distribution in patients with coronary artery disease [99]. Moreover, the most robust independent relationship between regional replacement fibrosis and MVP was found in the regions next to the posteromedial PM [98]. In addition, T1 mapping can be used for widespread fibrosis and tissue characterization. However, using only LGE might underestimate the myocardial inclusion. Indeed, a recent study found that 81% of patients without LGE on CMR and more than 80% of patients with MVP and MAD had ECV that included the mitral prolapse’s insertion point and were over the upper limit of normal [100].

Although CMR is not ideally suited to evaluating VHD, it can be used to evaluate valve defects comprehensively. The main strength of CMR lies in the highly accurate and reproducible assessment of ventricular volumes and function in patients with left- or right-sided valve disease [101,102]. In addition, the ability to image in unlimited planes is essential in patients with right-sided valve disease, which echocardiography poorly evaluates. Furthermore, quantifying RF and RVol with CMR is a particularly up-and-coming area [101].

Indeed, phase-contrast (PC) CMR velocity encoding is used for velocity measurements based on the accumulated phase of moving protons. Therefore, it is used to quantify the severity of valvular regurgitation and stenosis.

Even though CMR has long been the gold standard for determining chamber volumes, assessing their functionality, and evaluating the effects of severe MS (such as atrial and right ventricular dilation and dysfunction), studies have repeatedly shown that the relatively low temporal resolution of CMR causes mitral velocities to be considerably lower than those obtained from TTE [103]. In cases of MS, several thin sagittal slices taken from two orthogonal views perpendicular to the MV and tracing the smallest diastolic area at the leaflet tips are also possible [104]. The main limitation is represented by the frequent correlation of MS with atrial fibrillation (AF), which causes artifacts.

In the setting of MR, besides the precise quantification of LV and LA volumes, CMR has benefits in terms of precision and reproducibility for assessing the extent of LV remodeling and its impact on MV tethering, as well as for quantifying the grade of local or diffuse LV fibrosis and LV deformation or strain [105,106]. In addition, it helps understand the etiology of the underlying pathological process, especially in secondary MR. Moreover, CMR provides better spatial resolution, which aids in the detailed characterization of MV morphology and subvalvular apparatus [49]. It also quantifies MV RVol (MRV) using direct methods based on the velocity-encoded sequences and indirect quantifications based on the various SV calculations using the volumetric method or PC imaging [106]. Several prior studies have demonstrated the high accuracy of the MR fraction analysis (calculated by the formula MRV/LV SV × 100%), derived as the difference between the LV SV estimated by endocardial segmentation of cine pictures imaging and forward aortic flow volume [107].

Similarly, in patients with TR, CMR also helps in tissue characterization, providing information about myocardial impairment and RV fibrotic remodeling (using LGE, T1 mapping, and ECV quantitation) [108]. In addition, CMR enables the detection of RV strain and regional wall motion anomalies, albeit their current usefulness for TR is debatable [109]. Nevertheless, this method gives more accurate information about RV remodeling in athletes with TR. In addition, it provides three alternative techniques to accurately determine the reference SV (PC imaging from the pulmonic valve or aortic valve and volumetric LV SV) [78]. Although less proven, indirect CMR techniques are frequently used in the TR to calculate the RVol from the difference between the RV total and the forward SV through the pulmonary valve (using planimetry of short-axis cine pictures and PC velocity mapping, respectively) [110]. Patients at higher mortality risk were determined by a RF >50% and an RVol of >45 mL [110]. However, arrhythmias and transvalvular pacemaker leads are two examples of CMR’s limitations frequently found in patients with TR.

### 3.6. Cardiac Computed Tomography

CCT is another diagnostic method that can be used to evaluate CAs. CCT is more frequently applied to athletes at risk for SCD when there is a suspicion of coronary artery abnormalities [22]. In addition, particularly in master athletes, CCT may allow the identification and characteristic determination of coronary plaques [22]. However, CCT can also be indicated for evaluating valve diseases in athletes, not only in those with contraindications for CMR. Indeed, the evaluation of ventricular sizes, SV, EF, and cardiac mass demonstrated a marvelous correlation with data obtained by CMR [111].

In addition, CCT utility in valve disease assessment has been widely studied and has some specific applications. For example, in the case of MS, the CCT provides a clear depiction of MV leaflet thickening and calcification (because of high X-ray attenuation), evaluation of the extent of mitral annular calcification (which is rare in athletes), and accurate annular sizing [112]. Furthermore, Nazari et al. evaluated the performance of the CCT in diagnosing the MVP, reaching a sensitivity and specificity of 68.4% and 95.2% in individuating a 2.5 mm leaflet billowing [113]. Additionally, a recent study demonstrated that MAD is a relatively typical occurrence in normal adult hearts, with a bimodal distribution principally including the P1 and P3 scallops of the posterior mitral leaflet [114]. However, a few significant limitations of CCT for assessing MVP include exposure to radiation, inadequate temporal resolution, and the inability of tissue characterization. Indeed, the additional potential of CCT for risk assessment in MVP patients is still to be determined.

However, CCT, despite a lower temporal resolution than CRM, can analyze MR, provide precise volumetric measurements of chamber dimensions, and evaluate the MV and MA geometry. Moreover, pre-procedural planning using CCT is crucial for percutaneous MV replacement operations [106].

CCT can also provide morphological information about the TV considering its optimal spatial resolution and ability for multiplanar reconstructing [115]. The strengths of CCT imaging in TV settings, especially in pre-procedural planning, include evaluating the TA geometry, perimeter, and diameters and identifying the proper coronary artery position and course along with its distance from the TA [78]. However, the clinical role of CCT in evaluating athletes’ valves still needs further studies.

## 4. Atrioventricular Valve Disease in Athletes

### 4.1. Mitral Valve Prolapse

In 1963, Barlow first described the MVP, reporting the common association of mid–late systolic clicks with late-systolic murmurs [116,117]. MVP is one of the more frequent structural abnormalities of the heart and is more frequent in tall and female athletes, with a reported prevalence in the athlete’s population as similar to those reported in the general population (1–3%) [2,116,118].

Histologically, MVP is characterized by fibro-myxomatous changes in the MV leaflets [6]. The condition is diagnosed by evidence of > 2 mm systolic displacement of one or both MV leaflets beyond the annulus within the LA in end-systole [119]. MVP can be classified into primary and secondary (i.e., syndromic) when it co-occurs with connective tissue disorders [116].

MVP is one of the most challenging valve abnormalities in the athlete population. In 1966, Hancock et al. described that features associated with MVP were predominantly female sex, low or inverted T waves in leads II, III, and aVF, prolonged Q-T interval, syncope, or SCD [120].

Although most patients have a benign natural history, a small proportion may develop numerous cardiac complications, including severe MR due to degenerative disease or chordal rupture, infective endocarditis, embolic cerebrovascular accident, arrhythmias, and SCD [13,116]. In particular, in the context of MVP, SCD may occur because of chordal rupture or VAs [121]. In 1972, Sloman et al. reported the possibility of revealing myocardial irritability through exercise stress. Indeed, it was hypothesized that the existence of cardiomyopathy was associated with MVP [122]. Furthermore, athletic training can expedite degenerative processes and a propensity for arrhythmias or even SCD [13,116]. According to a cardiac pathology registry from the Veneto region in northeast Italy, the estimated annual SCD rate for MVP ranges from 0.2% to 0.4%, being MVP responsible for 7% of SCDs in young adults [86].

Athletes with MVP without significant regurgitation generally may not experience any symptoms. Instead, the condition is often an incidental finding on cardiac auscultation of a mid-to-late systolic click at the apex or detected on screening echocardiography [123]. However, when symptoms occur, athletes may experience dyspnea on exertion, reduced exercise tolerance, palpitations, fatigue, pre-syncope, syncope, and cerebrovascular accidents [123].

A high degree of valve regurgitation in MVP is widely acknowledged as a significant factor contributing to the incidence of SCD; nevertheless, the detection of MVP in victims of SCD or those who have survived life-threatening VAs indicates that there may be a connection between MVP without hemodynamic complications and arrhythmic SCD [124].

The histopathological investigation in 43 young patients (<40 years) with MVP and SCDs [86] revealed LV scarring at the level of the PMs with an adjacent free wall in all cases, and 88% also had scarring at the inferobasal wall, indicating that excessive stretch by the flail leaflets causing myocardial scarring promotes arrhythmogenesis [86]. Abnormal repolarization gradient, leading to the frequent observation of inverted T waves in this group of patients, may be caused by fibrosis in these areas [124].

In MVP, LV fibrosis is closely linked to the MV apparatus’s excessive mobility and the myocardium’s systolic stretch, which are both associated with MAD and systolic curling of the posterior MV leaflet (i.e., an unusual systolic motion of the posterior MA on the adjacent myocardium) [95]. In addition, the mid-systolic click may be caused by sudden tension resulting from systolic curling [86].

Specifically, MAD is an abnormal insertion of the hinge point of the posterior mitral leaflet on the atrial wall valve away from the ventricular myocardium, characterized by a clear separation of the basal portion of the posterolateral LV myocardium and the MA-left atrial wall [125]. MAD is associated with fibrosis of the LV close to the MA, supporting the arrhythmic role of this abnormality [124]. MAD’s dimension relates to prolapse severity, myocardial fibrosis, and VAs [126]. A TTE cut-off of MAD of 8.5 mm with suitable sensitivity and specificity for the prediction of non-sustained ventricular tachycardia was proposed by Carmo et al. [126].

As a result, VAs in MVP are due to myocardial fibrosis, marked leaflet redundancy, and MAD [95,127]. In a study by Caselli et al., athletes with MVP and VAs, compared to those without VAs, had a bigger LV and LA size, higher systolic BP, and were slightly older. Additionally, this group had a higher frequency of MAD (16% versus 3%; *p* < 0.001) [128].

### 4.2. Mitral Valve Regurgitation

MR is another category that should be considered in the athlete population. Indeed, MR is a common valvular disease and ranks as the third most prevalent form of VHD worldwide and second in Europe [27,118].

From a pathophysiological point of view, MR can be caused either by a primary abnormality of the mitral valvular apparatus (leaflets, chordae tendineae, PMs, or annulus) or secondary to non-valvular myocardial pathology (e.g., coronary artery disease, LA enlargement, or DCM because of tethering of the mitral leaflets and restricted leaflet course) [2,62].

The etiology of primary MR includes rheumatic heart disease, infective endocarditis, DCM, connective tissue disease (such as Marfan syndrome), and ischemic heart disease; however, the leading cause of MR among athletes is MVP [1,62]

Although uncommon, acute severe MR can manifest among CAs [2]. The primary causes of this condition include acute PM rupture, which can be triggered by intense isometric activity, and acute LV dysfunction due to ischemic heart disease or fulminant myocarditis [2]. When acute severe MR does occur, it often presents as acute decompensated heart failure, which requires urgent surgical intervention [2].

Generally, two main types of secondary MR are VFMR and atrial functional MR (AFMR). The VFMR occurs in dilated ventricles as a consequence of the increased distance between the displaced PM and MA, which leads to tethering (with a “seagull sign” of anterior MV leaflet), limited systolic leaflets motion, and a reduced coaptation height [129,130]. Contrarily, in the AFMR, caused by an enlarged LA, LV typically has preserved geometry and function or mildly reduced longitudinal strain [131]. Additionally, there is MA dilation (anteroposterior diameter > 35 mm in parasternal view or systolic annular diameter/diastolic anterior leaflet length ratio > 1.3), a counterclockwise-directed torque of the anterior annulus, a reduction in the posterior MV leaflet area, and relocation of the posterior annulus alongside the LA wall. This leads to decreased coaptation area with a coaptation point on the annular plane, and MV leaflets are generally flattened or mildly tethered [132].

Chronic MR prompts eccentric remodeling and hypertrophy of the LV in response to the increased volume resulting from rapid early diastolic filling; however, over time, this initial compensatory response may progress into maladaptive pathology, culminating in progressive LV dilatation [2]. Remarkably, both athletic training and MR may be associated with an enlarged LV cavity; however, an enlarged LV disproportionate to the level of exercise may suggest severe MR and an indication to refrain from competitive or leisure sports involving moderate- or high-intensity exercise [6].

Fábián et al. conducted a retrospective cohort study on 423 healthy athletes, including 42 (9.9%) with mild MR [4]. They found that exercise training can cause abnormal remodeling of the atrioventricular annuli beyond mere cardiac chambers enlargement. Athletes had greater MA area and tenting volumes than controls, particularly those with MR (8.2 ± 1.0 vs. 7.2 ± 1.0 cm^2^/m^2^, *p*-value < 0.05) [4]. In addition, athletes with MR had similar MA saddle shape parameters to non-athletes counterparts, suggesting insufficient geometrical adaptation to exercise; in contrast, those without MR had a more pronounced MA saddle shape [4].

On the other hand, LV’s physiologic hypertrophy can decrease the prevalence and severity of MR [20]. Indeed RWT, calculated as the ratio of ventricular septal wall thickness plus posterior wall thickness to end-diastolic internal diameter, was a negative predictor of MR, meaning that as RWT increased, the odds of MR decreased [20].

When the regurgitant jet grows, progressively becoming severe, MR is accompanied by a raised atrial pressure and higher LV preload with an escalating dilation of the LV cavity [133]. Therefore, chronic MR causes eccentric LV remodeling, leading to maladaptation with persistent LV dilation, which can contribute to systolic dysfunction. In addition, concurrent LA enlargement diminishes atrial performance, raising the risk for AF [2].

There is a different contribution in the MR between CAs engaged in various sports. Small sizes and hypertrophy due to strength training preserve the mitral leaflet coaptation; at the same time, competitive endurance athletes have more frequent annular dilatation and enlarged LV, contributing to the PM’s outward and downward displacement of the MV edge. In the absence of cordae elongation, this feature leads to poorer coaptation and a greater prevalence of MR [7,134,135]. Nevertheless, it is crucial to remember that the physiologically dilated LV in some competitive endurance athletes, in the context of MR, may raise a concern of marked volume overload [1]. However, contrary to the spherical geometry in DCM, sports remodeling preserves the elliptical shape of the ventricle and dissuades the positive feedback system from further aggravation of the MR [136].

A LVEDD with a cut-off of 35 mm/m^2^ and 40 mm/m^2^ (for males and females, respectively) was shown to help identify athletes with primary MR with a clinically significant LV cavity increase [62]. However, more research is necessary since a LVEDD value greater than 60 mm suggests severe MR and may require a surgical MV repair [62]. In addition, these measurements can be deemed cautious, especially in male competitive endurance athletes with a body surface area <2 m^2^; therefore, a customized strategy that considers these characteristics is advised before placing exercise limitations [5]. Measures of LV pump function, including LVEF, tend to overstate actual myocardial performance because of the low impedance caused by regurgitation into the LA, which unloads the LV during systole [137]. LV dysfunction in the context of MR is defined as LVEF < 60% or LV end-systolic dimension >40 mm [2]. The distinction between LV dilation caused by athletic training versus that caused by severe MR is difficult when the LVEDD is <60 mm (or <40 mm/m^2^).

Athletes with MR should be evaluated yearly by a physical exam, Doppler echocardiogram, and exercise stress test that matches the physical demands of their respective sports [138].

### 4.3. Mitral Valve Stenosis

Primarily causes of MS are rheumatic or degenerative factors. Nevertheless, rheumatic valve disease remains a significant contributor to valve disease worldwide, with a prevalence of approximately 0.02–0.2% in developed countries [27,62].

Although rheumatic valve disease is rare in high-income countries, with the increasing globalization of elite sport and emigration patterns, cardiologists in the Western world may encounter rheumatic MS among individuals who aspire to exercise or athletes from the developing world [6]. In addition, the prevalence of rheumatic MS is significantly higher in women, accounting for approximately 80% of cases [53]. The principal mechanism of MS in rheumatic heart disease is represented by commissural fusion and thickening of the posterior leaflet [1]. In contrast, degenerative MS with mitral annular calcification is a distinct entity that typically affects elderly patients with other comorbidities [27].

The characteristic mid-diastolic rumbling murmur detected during auscultation can indicate the presence of MS [1]. Patients typically experience dyspnea, especially during exercise or in combination with conditions that increase HR or flow across the MV [139]. Although MS rarely causes SCD, exercise can lead to acute pulmonary edema due to marked pulmonary capillary and PAP surges [62]. It is unknown if exertion-induced pulmonary pressure affects the lungs and RV long-term or if intense exercise impacts the risk of developing AF [1].

The risk stratification of exercising individuals with MS is mainly based on symptoms and a detailed echocardiogram with a specific interest in the severity of the lesion and accompanying sPAP. In addition, the assessment should include a maximal exercise stress test to identify concealed symptoms and functional capacity. At the same time, most people with severe MS are usually sufficiently symptomatic during exercise and incapable of engaging in competitive sports involving moderate or high CV demand [62]. However, athletes with mild or moderate MS may be asymptomatic even after vigorous exercise [5].

The continuity equation method for echocardiographic defining of MS severity is recommended in patients with degenerative valvular or annular calcification as other parameters (such as the PHT-related method) will not be valid in case of noncompliant LA or LV. However, the benchmark method for evaluating MS is mitral valve area (MVA) measurement using planimetry, considering the difficulties in performing it. On the other hand, mean transvalvular gradient and sPAP reflect its effects and have a better predictive value [1]. An MVA is clinically significant when less than 1.5 cm^2^, translating to a mean transmitral gradient of 5 to 10 mm Hg at normal resting HRs [1,53]. However, this mean pressure gradient and LV filling rely on different factors. Indeed, transvalvular flow rate, diastolic filling time, and changes in HR caused by physical exertion or arrhythmia can exacerbate symptoms even when there is no sign of severe MS at rest [53].

The narrowing of the valve area occurs gradually, at a rate of 0.1–0.3 cm^2^ per year. Therefore, the risk of adverse events increases when patients develop limiting symptoms or substantial PH in the presence of severe MS [139].

### 4.4. Tricuspid Valve Regurgitation

TR may be due to acquired pathologies such as rheumatic fever, infective endocarditis, carcinoid syndrome, or congenital disease such as Ebstein’s anomaly. The rheumatic disease affects the TV in 20 to 30% of cases, often associated with mitral and aortic involvement. In general, it is a mild involvement that is difficult to appreciate in 2DE if particular attention is not paid to the search for valve leaflets and commissural fusion. In addition, rheumatic disease causes more frequent regurgitation than stenosis [1].

However, in most instances, TR occurs in conjunction or is functional secondary to the RV, RA, or TA dilation from other pathologies, including left-sided heart or valve diseases, AF remodeling, PH, or right ventricular dysfunction. Therefore, exercise limitations relate to the underlying pathology in most patients with secondary TR [27].

Primary VHDs show a similar prevalence in athletes as in the general population. However, athletes report TR more frequently than sedentary subjects, suggesting predominantly secondary functional origin [7]. For example, most athletes with functional MR have concomitant TR, and interestingly, the MR group is presented with higher TA dimensions as well. This phenomenon implies that athletes prone to more excessive dilation of the MA are susceptible to a more pronounced TA remodeling [4]. Of note, it has been suggested that alterations in TA geometry can be present even in the case of degenerative processes affecting the MA primarily [140].

Although the pathophysiological mechanism underlying this increased prevalence of TR in athletes is not yet fully understood, it is essential to underline that TR in athletes represents an adaptive mechanism secondary to the dilation of the RA and RV. During exercise, the RV is subject to a lower pressure overload than LV because the pulmonary resistance is lower than the systemic resistance [10]. However, like the left heart, an enlarged RV with diminished systolic function is a characteristic and unmistakable feature in high-level endurance athletes, inducing a phenomenon commonly known as “RV fatigue [141]”. Indeed, compared to non-athletic control subjects, global and regional RV systolic function at rest is mildly reduced in endurance athletes. These findings can be considered a “physiological” adaptation to intensive exercise [25].

In addition, during exercise, the increased LV CO determines an augmented venous return to the right chambers, with consequent progressive enlargement. This feature is associated with increased wall thickness and altered diastolic function for the increased atrial component of the flow pattern across the TV [25]. In addition, the increased volume load induces a significant change in the RV and RA size and TA diameter. RV enlargement of the RV will also impose a tethering effect via chordae tendinae on the annulus fibrosus that attaches the valvular system, leading to poorer adaptation of the valves and, thus, a higher prevalence of valvular regurgitations [3].

In front of a bilateral chambers dilation, the TA has more lipidic and less fibrotic characteristics than the MA, making it more likely to dilate [142]. As demonstrated by Arbab-Zadeh et al., the LV responded to the initiation of endurance training with an increase in mass without a volume change (concentric hypertrophy); an increase in LVEDV occurred only after 6 to 9 months of progressive training, restoring the baseline mass-to-volume ratio (eccentric hypertrophy). In contrast to the LV, the RV responded to endurance training with a proportional increase in mass and volume, thereby maintaining a constant mass-to-volume ratio (eccentric hypertrophy) at all activity levels [143]. This finding suggests that athletes report TR more frequently, confirming the predominantly secondary (functional) origin. Indeed, in a study by Gjerdalen et al. TR was significantly higher in athletes compared to controls (58% vs. 36%) [3].

As proof of EICR, a recent study proposed mid-diastolic tricuspid regurgitation (MDTR) as a possible novel echocardiographic marker of the athlete’s heart. MDTR is a physiological finding in a highly trained endurance athlete due to distinct sports adaptations. MDTR is observed between the passive early diastolic and active late diastolic ventricular filling, and the extent of regurgitation is dependent on intrathoracic pressure (increased during expiration and Valsalva maneuver). The authors postulated two possible mechanisms typical features of the athlete’s heart: sinus bradycardia, which determines an increased diastole time, and a dilated and compliant RV, which determines a direct transfer of intrathoracic pressure variations related to respiration [144].

Conditions that result in an increase in RV volume or pressure overload cause local remodeling of RV geometry, PM displacement (mostly the septal one), deformation of the spatial arrangement among chords, PMs, leaflets, TA enlargement, and its dynamics alteration, bringing to leaflet tethering and mal-coaptation with a VFTR [142]. Unlike VFTR, AFTR presents the shortest leaflet angle of tethering. TA dilatation (defined as >40 mm or >21 mm/m^2^ in the apical four-chamber view) is the main contributor to mal-coaptation and regurgitation, which, together with RA enlargement, precedes RV dilation [145]. Similarly, the annulus fibrosus is tethered by the chordae tendinae when the RV is enlarged in athletes owing to volume stress, which results in inferior adaptation and a higher prevalence of TR.

Indeed, Gjerdalen et al. [3] found that athletes had a greater prevalence of TR (58%) compared to controls and that RA areas were consistently larger in athletes with TR than those without. Furthermore, compared to controls, CAs have more significant TA areas and tenting volumes, with the TV annulus being more severely enlarged than the RV [4].

Due to their prognostic significance, TA enlargement, RV and RA measurements, and the performance of RV should all be assessed in secondary TR [146]. However, according to research by Prihadi et al., worse RV-free wall longitudinal strain, detected by speckle-tracking echocardiography, is related to worse outcomes in patients with severe TR and is more likely to reveal RV dysfunction [147].

### 4.5. Tricuspid Valve Stenosis

Isolated TS is rare in adults. Instead, in almost all cases, it is caused by rheumatic disease with concomitant MV involvement. In addition, carcinoid tumors can affect the right heart sections leading to TR or TS [1].

The 2D echocardiography is used to evaluate the TV’s anatomy utilizing a variety of windows, including the parasternal right ventricular inflow, the parasternal short axis, the apical four-chamber, and the subcostal four-chamber [148]. In 2DE, the valve leaflets’ thickening and shortening are evident. In addition, the fusion of the commissures and the domed morphology indicate a rheumatic cause. Finally, the Doppler recording of the transvalvular flow velocity allows the calculation of the mean pressure gradient [148].

Nevertheless, there are no generally accepted and standardized parameters for TS severity grading, even though some variables are indicative of hemodynamically significant TS, such as mean pressure gradient ≥ 5 mmHg at normal HR, inflow VTI > 60 mm, pressure half-time (T1/2) ≥ 190 ms and valve area by continuity equation ≤ 1 cm^2^ [27,148].

## 5. Eligibility for Sports

Principal recommendations according to valvular disease severity are resumed in Table 3.

### 5.1. Mitral Valve Prolapse

Given that the spectrum of disease for MVP can range from benign imaging finding to fatal VAs, there has been a recent interest in characterizing risk factors for arrhythmic MVP, including imaging features [2].

In particular, ventricular function and severity of MR by echocardiography, exclusion of fibrosis by CMR, in addition to electrocardiographic alterations, presence of arrhythmias, syncope, and patient history, make part of the most significant societies of cardiology’s criteria for competitive sports eligibility [149]. Generally, most physically active people with MVP and mild to moderate regurgitation, in the absence of alert criteria, can engage in all competitive sports. However, while MVP usually has an excellent prognosis and does not require sports restrictions without significant MR and VAs, identifying athletes at higher risk of SCD is crucial [112]. Despite the degree of regurgitation, arrhythmic SCD is uncommon (0.2–0.4% per year). However, several markers may signify an increased risk of SCD. Therefore, according to the ESC guidelines, symptomatic MVP patients should avoid recreational or competitive sports if they have any of the following high-risk features: bileaflet prolapse, T-wave inversion in the inferior leads on the 12-lead ECG, documented VAs, severe MR, long QT, LV systolic dysfunction, family history of SCD, fibrosis of the PMs or LV inferolateral basal wall [5,6,124]. In addition, caution should be used in individuals with an associated aortic cusp prolapse and/or aortic root dilation [150].

### 5.2. Mitral Valve Regurgitation

General recommendations regarding exercise and sports in athletes with MR should consider different aspects that can aid in determining the appropriate level of physical activity and help in decision-making. These include symptomatic status, MR severity, LV dimensions and function, estimation of sPAP at rest and during exercise, functional capacity, myocardial reserve, and the presence or absence of arrhythmias [1,6,62].

Athletes with mild MR are encouraged to participate in all competitive sports (class of recommendation I, level of evidence C) [6], and an annual assessment should be prompted [150].

Athletes with moderate MR should be considered (class of recommendation IIa, level of evidence C) for all competitive sports if they are asymptomatic with a suitable functional capacity, provided LVEDD is <60 mm or <35.3 mm/m^2^ in men and <40 mm/m^2^ in women, LVEF is ≥60%, resting sPAP is <50 mmHg, in sinus rhythm, and exercise test is normal without complex arrhythmias [1,6]. Athletes with moderate MR should undergo a 6-month echocardiographic follow-up, according to the Italian Guidelines for Management of Athletes [150].

Athletes with severe MR may be deemed suitable (class of recommendation IIb, level of evidence C) for low-intensity sports if they are asymptomatic with a suitable functional capacity, LVEDD is <60 mm or <35.3 mm/m^2^ in men and <40 mm/m^2^ in women, LVEF is ≥60%, resting sPAP is <50 mmHg and exercise test is normal without complex arrhythmias [1,6]. Athletes with moderate MR should undergo a 6-month echocardiographic follow-up, according to the Italian Guidelines for Management of Athletes [1,6].

Therefore, individuals with LVEF < 60%, symptomatic MR, and reduced exercise capacity or individuals with MR with exercise-induced complex arrhythmias should not participate in competitive or leisure sports. Finally, individuals on long-term anticoagulation therapy for AF should not engage in contact/collision sports [1,6].

The indications for surgical intervention in athletes are similar to those in the general population, and surgical MV repair performed by an experienced surgeon is preferred over MV replacement [2]. After MV repair surgery, athletes’ eligibility to participate in sports should be evaluated by experts to exclude VAs and residual MR at rest and during exercise [123,150].

### 5.3. Mitral Valve Stenosis

For patients with MS who want to participate in competitive sports with minimal or no symptoms, it is recommended to undergo exercise stress testing to evaluate their ability to handle the activity required for their sport [62]. In addition, ESE offers supplementary data by assessing alterations in the mitral gradient and PAP [1].

Guidelines suggest that individuals with a mild MS (MVA 1.5–2.0 cm^2^) can engage in all competitive sports (class of recommendation I, level of evidence B) if they are asymptomatic, in sinus rhythm, have a resting sPAP < 40 mmHg, and have a normal exercise test [6].

Athletes with moderate MS (MVA 1.5–1.0 cm^2^) may participate in low-intensity competitive sports (class of recommendation IIb, level of evidence C) if they are asymptomatic, in sinus rhythm, have a resting sPAP < 40 mmHg, and have a normal exercise test [6].

Instead, individuals with severe MS (MVA < 1.0 cm^2^) should avoid participating in any competitive sports (class of recommendation III, level of evidence C) [6]. Indeed, mildly symptomatic individuals with severe MS may only participate in mild-intensity leisure activities.

Furthermore, those with MS of any severity who develop AF should be long-term anticoagulated and avoid any competitive sports involving physical contact [5,62]. Finally, symptomatic individuals with severe MS should be referred for percutaneous mitral commissurotomy or valve replacement if they have unfavorable anatomy [1]. After surgical treatment, athletes can return to sports if they are asymptomatic and have evidence of suitable hemodynamic valve function [1]. In cases of balloon mitral valvuloplasty with suitable results (i.e., MVA > 2.0 cm^2^), regular exercise and competitive sport may be considered in asymptomatic individuals with suitable functional capacity.

### 5.4. Tricuspid Valve Regurgitation

In CAs, echocardiographic evaluation of sPAP is vital among the criteria for sports eligibility. However, it is crucial to remember that with greater severity of TR, higher resting RA pressures, and diminished RV function, rapid equilibration of the RV and RA pressures ensues, making it impossible to estimate correctly sPAP [151].

In most patients with secondary TR, exercise limitations relate to the underlying pathology. Individuals with any degree of TR, with a resting sPAP > 50 mmHg at rest, and RV dysfunction may participate in low-intensity sports only. Also, individuals with right atrial pressure > 20 mmHg and any degree of TR should avoid competitive sports [1,6].

Nevertheless, individuals with mild TR, without the above-mentioned limitations, can participate in all sports. Certainly, mild TR is common in athletes and accompanied by physiological dilatation of the inferior vena cava, which is easily collapsible with inspiration [6].

Individuals with mild TR, without the above-mentioned limitations, are eligible for all levels of exercise [1].

Individuals with moderate TR, without the above-mentioned limitations, are eligible for all sports disciplines of any intensity if also they have a normal biventricular systolic function, normal exercise testing, and sPAP is <40 mmHg at rest [1].

Finally, individuals with severe TR, without the above-mentioned limitations, are eligible for only low-intensity exercises [1].

### 5.5. Tricuspid Valve Stenosis

Athletes with mild TS are eligible for all sports if they are asymptomatic and have a normal ventricular function; however, individuals with moderate or severe stenosis should avoid sports [1]. Finally, in athletes with coexisting MS and TS, recommendations should be based on the severity of MS.

## 6. Conclusions

Atrioventricular valve diseases are more frequently experienced by athletes than non-athletes, suggesting predominantly functional origin. Indeed, adaptation to exercise determines several changes, including atrioventricular remodeling. Therefore, assessing and interpreting athletes with valve pathology requires special attention since the pathologic mechanisms may differ from inactive individuals. In addition, the differential diagnosis of some cases of cardiac chambers dilation can be problematic in the presence of regurgitation.

In this complex scenario, the possible imaging modalities in athletes are several, including quantifying direct and indirect signs of atrioventricular valve disease at rest or during exercise. Nevertheless, these techniques provide an accurate, objective, non-invasive, and reproducible method for MV and TV assessment. Furthermore, they help clarify the etiologic mechanism, the EICR contribution to regurgitation, the identification of athletes at higher risk of arrhythmias and SCD, and eventually indicate whether athletes are eligible for participation in competitive sport.

## Figures and Tables

**Table 1:** Role of multimodality imaging in the assessment of VHD in athletes. The complementary part of other imaging techniques to standard 2D echocardiography.

	3D Echo	Stress Echo	MW	CMR	CCT
MVP	Identification of leaflet billowing, prolapse, and flail; characterization of MAD.	Evaluation of sPAP increase during exercise.	Insight into myocardial contraction.	Fibrosis detection.	Characterization of mitral valve apparatus anatomy.
MR	Valve apparatus morphology and quantification of regurgitation.	Evaluation of hemodynamic consequences and arrhythmias during exercise.	Capacity to reflect “true” EF.	Tissue characterization for insight into LV remodeling.	Assessment of MV geometry; cardiac chambers measurement.
MS	Valve apparatus morphology and assessment of MVA.	Symptoms, sPAP, and MV dynamic gradients evaluation during exercise.	Information about LV impairment.	Evaluation of MVA.	Assessment of leaflet and annular calcification; cardiac chambers measurement.
TR	RV sizing measurement with calculation of 3D RVEF.	Symptoms, sPAP, and TV dynamic gradients evaluation during exercise.	Identification of earlier modifications in RV contractility.	Tissue characterization for insight into RV remodeling.	Assessment of leaflet and annular calcification; cardiac chambers measurement.
TS					

Abbreviations: CMR: cardiac magnetic resonance; EF: ejection fraction; LV: left ventricle; MAD: mitral annular disjunction; MR: mitral regurgitation; MS: mitral stenosis; MV: mitral valve; MVA: mitral valve area; MVP: mitral valve prolapse; MW: myocardial work; PH: pulmonary hypertension; RV: right ventricle; RVEF: right ventricular ejection fraction; sPAP: systolic pulmonary artery pressure; TR: tricuspid regurgitation; TS: tricuspid stenosis; TV: tricuspid valve; VHD: valvular heart disease.

**Table 2:** Most frequently observed differences and similarities in multimodality imaging assessment of mitral and tricuspid valve disease severity between athletes and the general population.

		General Population				Athletes			
Imaging Parameters		*Mitral Valve*		*Tricuspid Valve*		*Mitral Valve*		*Tricuspid Valve*	
		MS	MR	TS	TR	MS	MR	TS	TR
TTE/ CRM	Origin	Primary rheumatic	Primary and secondary	Primary	Primary and secondary	Primary rheumatic	Mixed FR *	Primary	Mixed FR *
	LV size	n. **	n./↑/↑↑	n.	n.	↑ ***	↑/↑↑	↑	↑
	LVEF	n./↓	↓ ****	n.	n./↓	n.	n.	n.	n.
	RV size	n./↑	n./↑	n./↑	n./↑/↑↑	↑	↑	↑	↑/↑↑
	RV function	n./↓	n./↓	n./↓	n./↓	n./↓ transitory	n./↓ transitory	n./↓ transitory	n./↓ transitory
	LA size	↑↑	n./↑/↑↑	n.	n.	↑↑↑	↑/↑↑	↑	↑
	RA size	n.	n.	↑↑	n/↑/↑↑	n./↑	n./↑	↑↑↑	↑/↑↑
	VC		≥7 (≥8 mm)		>7 mm		=^¶^		=
	PISA		>9 mm		>9 mm		=		=
	EROA		≥40 (or 30 mm^2^)		≥40 mm^2^		≥30 mm^2^		=
	RVol		≥60 mL (or 45 mL)		≥45 mL		=		=
	RF		≥50%		≥50%		=		=
	Valve area	≤1.5 cm^2^		≤1 cm^2^		=		=	
	PHT	≥150 ms		≥190 ms		=		=	
	sPAP	>50 mmHg			n./↑	>> ^ 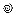 ^			↑/↑↑
	Mean gradient	>10 mmHg		≥5 mmHg		=		=	
3D Echo	VCa/qEROA ^#^				≥75 mm^2^				=
	Valve morphology/ leaflet motion	Commissural fusion	Tethering n./↑/↑↑	Leaflets’ thickening	TA↑	=	↑Saddle-shaped, ↑ tenting	=	TA↑↑
Stress Echo	Mean gradient	≥15 mmHg				=			
	sPAP	>60 mmHg	≥60 mm Hg		45 mmHg	>>	>>		55–60 mmHg
GLS and MW Parameters		GLS↓↓	↓GWW		↓GCW if HF	GLS n./↓	n. GWW		GCW n./↑

* Mixed atrial and ventricular functional regurgitation; ** normal values; *** increased; **** decreased; ^¶^ no differences; 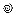 higher values; ^#^ 3D VC area or quantitative EROA 95–114 mm^2^ for massive TR and ≥115 mm^2^ for torrential TR; Abbreviations: CMR: cardiac magnetic resonance; EROA: effective regurgitant orifice area; FR: functional regurgitation; GLS: global longitudinal strain; GCW: global constructive work; GWW: global wasted work; HF: heart failure; LA: left atrium; LV: left ventricle; LVEF: left ventricular ejection fraction; MR: mitral regurgitation; MS: mitral stenosis; MW: myocardial work; PHT: pressure half-time; PISA: proximal isovelocity surface area; RA: right atrium; RF: regurgitant fraction; RV: right ventricle; RVol: regurgitant volume; sPAP: systolic pulmonary artery pressure; TA: tricuspid annulus; TR: tricuspid regurgitation; TS: tricuspid stenosis; TTE: transthoracic echocardiography; VC: vena contracta.

**Table 3:** Exercise restriction in competitive athletes considering VHD severity.

Valvular Heart Disease	Disease Severity	Recommendation
Mitral Valve Prolapse	Mild to moderate MR or no alert criteria ^1^	No restriction. All levels of exercise.
	Alert criteria ^1^ or severe MR	Restriction. Low-intensity sports if suitable.
Mitral Regurgitation	Mild	No restriction. All levels of exercise.
	Moderate	No restriction if suitable. ^2^ All levels of exercise.
	Severe	Restriction. Low-intensity sports if suitable. ^2^
Mitral Stenosis	Mild	No restriction if suitable. ^3^ All levels of exercise.
	Moderate	Restriction Low-intensity sports if suitable. ^3^
	Severe	Restriction Avoid all competitive sports.
Tricuspid Regurgitation	Mild	No restriction if suitable. ^4^ All levels of exercise.
	Moderate	No restriction if suitable. ^4,5^ All levels of exercise.
	Severe	Restriction. Low-intensity sports if suitable. ^4^
Tricuspid Stenosis	Mild	No restriction if suitable. ^6^ All levels of exercise.
	Moderate	Restriction. Avoid all competitive sports.
	Severe	Restriction. Avoid all competitive sports.

^1^ High-risk alert features for MVP: bileaflet prolapse; T-wave inversion in the inferior leads; documented VAs; severe MR; long QT; LV dysfunction; family history of SCD; fibrosis of the papillary muscles or the LV inferolateral basal region. ^2^ LVEDD < 60 mm or < 35.3 mm/m^2^ in men and <40 mm/m^2^ in women; LVEF ≥ 60%; sinus rhythm; resting sPAP < 50 mmHg; normal exercise test. ^3^ Asymptomatic; sinus rhythm; sPAP < 40 mmHg; normal exercise test. ^4^ Individuals with any degree of TR, sPAP > 50 mmHg at rest, and RV dysfunction may participate in low-intensity sport only. Individuals with a right atrial pressure of more than 20 mmHg and any degree of TR should avoid competitive sports. ^5^ Normal biventricular systolic function; normal exercise testing; sPAP < 40 mmHg at rest. ^6^ Asymptomatic; normal ventricular function; in athletes with coexisting MS and TS, recommendations should be based on the severity of MS. The indications have been extrapolated from van Buuren et al. [1] and Pelliccia et al. [6].

## Data Availability

Not applicable.

## Abbreviations

The following abbreviations are used in this manuscript:

**Abbreviation**	**Definition**
AF	Atrial fibrillation
AFTR	Atrial functional tricuspid regurgitation
BP	Blood pressure
CAs	Competitive athletes
CCT	Cardiac computed tomography
CMR	Cardiac magnetic resonance
CO	Cardiac output
CV	Cardiovascular
DCM	Dilated cardiomyopathy
ECV	Extracellular volume
EICR	Exercise-induced cardiovascular remodeling
ESE	Exercise stress echocardiography
GCW	Global constructive work
GLS	Global longitudinal strain
GWE	Global work efficiency
GWI	Global work index
GWW	Global wasted work
HCM	Hypertrophic cardiomyopathy
HR	Heart rate
LA	Left atrium
LGE	Late gadolinium enhancement
LV	Left ventricle
LVEDD	Left ventricle end-diastolic diameter
LVEDV	Left ventricle end-diastolic volume
LVEF	Left ventricular ejection fraction
LVOT	Left ventricular outflow tract
MA	Mitral annulus
MAD	Mitral annular disjunction
MDTR	Mid-diastolic tricuspid regurgitation
MR	Mitral regurgitation
MS	Mitral stenosis
MV	Mitral valve
MVA	Mitral valve area
MVP	Mitral valve prolapse
MW	Myocardial work
PAP	Pulmonary artery pressure
PC	Phase-contrast
PISA	Proximal isovelocity surface area
PMs	Papillary muscles
PSL	Pressure-strain loop
RA	Right atrium
RV	Right ventricle
RVEIO	Right ventricle early inflow–outflow
RF	Regurgitant fraction
RVol	Regurgitant volume
RVOT	Right ventricular outflow tract
RWT	Relative wall thickness
SCD	Sudden cardiac death
SPAP	Systolic pulmonary artery pressure
SV	Stroke volume
TA	Tricuspid annulus
TR	Tricuspid regurgitation
TS	Tricuspid stenosis
TV	Tricuspid valve
TTE	Transthoracic echocardiography
VAs	Ventricular arrhythmias
VC	Vena contracta
VFTR	Ventricular functional tricuspid regurgitation
VHD	Valvular heart disease
VTI	Velocity time integral
2DE	Two-dimensional echocardiography
3DE	Three-dimensional echocardiography
